# Allergenic Properties and Molecular Characteristics of PR-1 Proteins

**DOI:** 10.3389/falgy.2022.824717

**Published:** 2022-02-08

**Authors:** Andrea Wangorsch, Stephan Scheurer, Miguel Blanca, Natalia Blanca-Lopez, María Luisa Somoza, Laura Martín-Pedraza

**Affiliations:** ^1^Molecular Allergology, Paul-Ehrlich-Institut, Langen, Germany; ^2^Foundation for Biomedical Research and Innovation, Hospital Universitario Infanta Leonor, Madrid, Spain; ^3^Allergy Service, Hospital Universitario Infanta Leonor, Madrid, Spain

**Keywords:** pathogenesis-related protein-1, PR-1, allergen, occupational allergy, allergy diagnosis

## Abstract

Only a small fraction of proteins in plants and animals are classified as allergens. The allergenic properties are frequently attributed to certain functional characteristics of the proteins, such as a role in the plant defense against biotic and abiotic stress, to achieve the systematic acquired resistance. In line with this, eight members out of 17 functional pathogenesis-related (PR) protein families have been characterized as allergens. The present review summarizes the molecular features and allergenic significance of allergens of the PR-1 family. Not many allergens have been identified as belonging to this protein family, with most of them having a pollen origin, like mugwort or Bermuda grass. Molecular and structural features of allergenic PR-1 proteins are discussed and attributed to their IgE-reactive properties, clinical manifestation, and cross-reactivity among different foods and inhalants.

## Introduction

Several allergens have been identified as members of the pathogenesis-related (PR) protein families, PR-2 (ß-1-3 Glucanases), PR-3 and PR-4 (Chitinase), PR-5 (Thaumatin-like), PR-8 (Chitinase), PR-10 (Bet v 1-like), PR-14 (nsLTPs) (1, 2), and from the PR-1 family (3). The PR proteins of the same family are known to have highly-homologous sequences and similar functions (4).

The classification of PR-1 proteins is not consistent. On one hand, PR-1 proteins were classified as either cysteine-rich secretory proteins (CRISP) or antigen 5 (Ag5) type proteins (4). These proteins either belong to sperm coating protein (SCP)-like extracellular protein family, PR-1 protein family, or allergen V5/Tpx-1-related protein family (4), as part of the SCP/TAPS (Tpx-1/Ag5/PR-1/Sc7) protein superfamily (5, 6). In another classification, PR-1 proteins are described as members of a CAP protein superfamily, encompassing the Cysteine-rich secretory proteins in vertebrates, Antigen 5 in insects, and PR-1 in plants (7–9). Members of this superfamily are found across the bacterial, fungal, plant, and animal kingdoms, and they have broad physiological functions (10). Combining both classifications, the PR-1 protein family sometimes is termed as CAP/SCP/TAPS superfamily (9).

**Figure 1:**
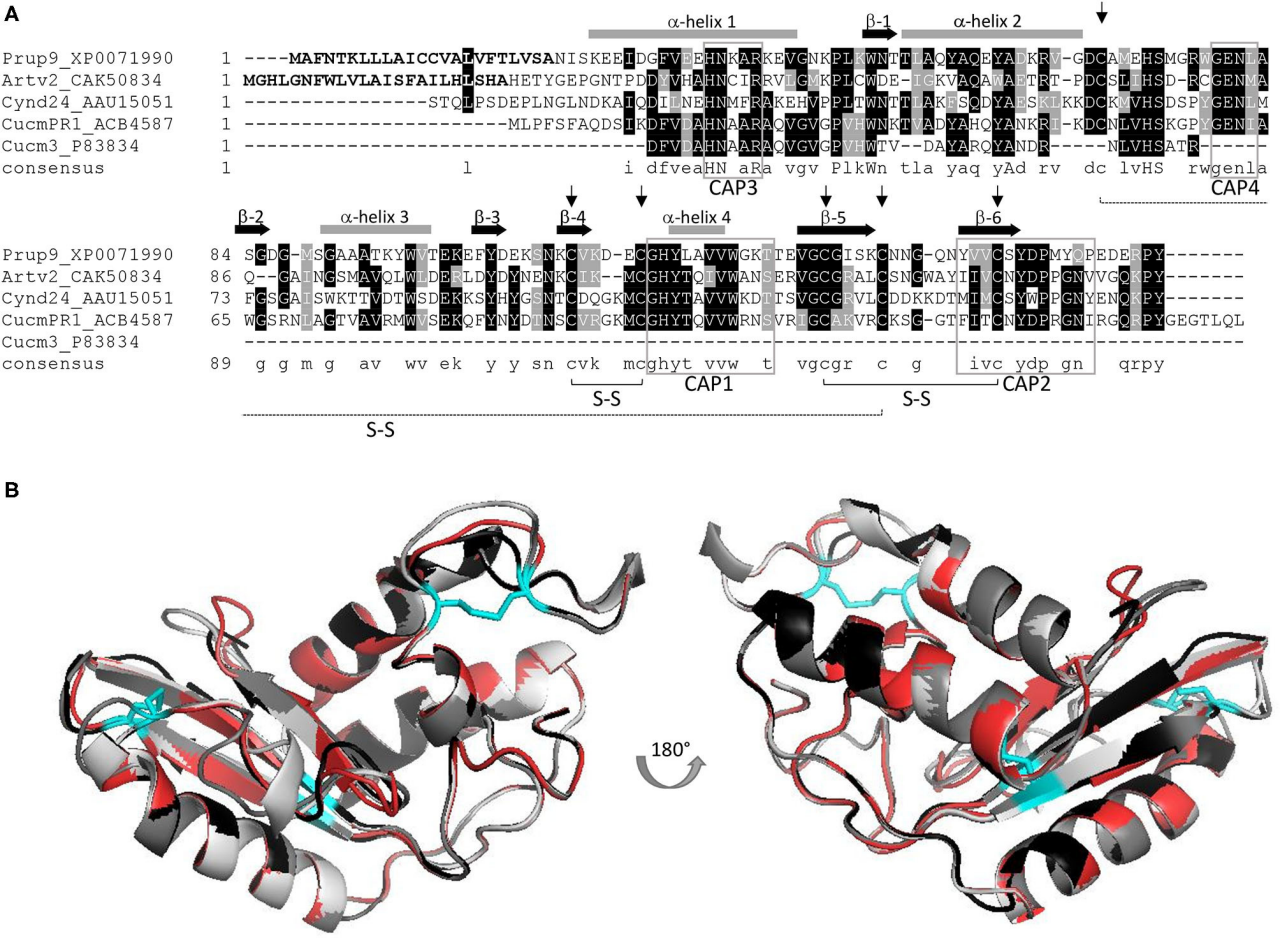
**(A)** AA alignment of PR-1 allergens Pru p 9, Art v 2, Cyn d 24, and Cuc m 3, including the full-length sequence of a Cuc m PR-1 protein; signal peptides of Pru p 9 and Art v 2 are displayed in bold; six conserved cysteins are indicated by an arrow, and the corresponding S-S bonds are outlined; CAP signature motifs 1–4 are marked (gray box) and α-helix (gray bar) and β-sheets (black arrow) are included; consensus sequence: conserved aa: capital letters, similar aa: small letters, no similarity: no letter; Figure adapted from (7). **(B)** Superimposition of 3D models including Pru p 9 (red), Art v 2 (black), Cyn d 24 (gray), and Cuc m PR1 (light gray). Disulfide bonds are indicated in cyan. All proteins were modeled using Swiss model and P14a from *Lycopersicon esculentum* (PDB-1CFE) as reference.

Pathogenesis-related-1 proteins were initially identified in tobacco plants in response to TMV infection (11). They are ubiquitously abundant, with a role in plant growth and flowering, in cell wall loosening, and with a function in biotic and abiotic stress responses (8, 12). PR-1 expression is induced by salicylic acid signaling, but the molecular mechanism to achieve disease resistance is not known (8). Domain analysis indicated that most PR-1 proteins have a conserved α*βα* sandwich folded CAP domain of 150 amino acids (4, 9). The CAP domain comprises the whole PR-1 protein and contains four conserved signature motifs (CAP1-4), with six specific cysteine residues encompassing 3 disulfide bonds (Figure 1A) (10) and is attributed to ion-, fatty acid, and sterol-binding capacity (8, 9, 13). Frequently PR-1 proteins are secreted as 14–17 kDa glycoproteins (9), and are classified as acidic or basic PR-1 (8), which are stabilized by hydrophobic interaction, hydrogen bonds, and intramolecular disulfide bonds. In line with this, PR-1 proteins are considered as highly stable proteins (14). The size, stability, and resistance to proteases, along with hydrolytic and membrane permeabilizing ability, have been claimed to make PR-proteins, including PR-1 proteins, excellent candidates to elicit an allergenic response (15). A summary of the PR-1 allergens described in this review is shown in Supplementary Table 1.

## PR-1 Allergens

### Cuc m 3, a PR-1 From Melon

Cosensitization to melon (*Cucumis melo*), belonging to the *Cucurbaitaceae* family, has been reported in the pollen food allergy syndrome, in particular in ragweed and grass pollen allergic patients (16, 17). In line with this, occupational respiratory allergy to melon flowers and fruit has been described (18).

So far three melon allergens are described: Cuc m 1 (subtilisin-like-protease), Cuc m 2 (profilin), and Cuc m 3. Cuc m 3.0101 (GenBank# P83834,) was the first PR-1 protein characterized as an allergen (19). Initially, a stretch of 41 amino acids (aa) was identified by the N-terminal sequencing of internal peptides of the 17 kDa protein derived from melon juice. A total of 12 out of 17 melon-allergic individuals with positive skin prick test (SPT) to melon extract, melon specific IgE >0.94 kU/L, and double-blind placebo-controlled food challenge (DBPCFC)-confirmed melon allergy showed IgE-binding to purified Cuc m 3. However, the frequency of IgE-sensitization (71%) was not in concordance with a low frequency of positive SPT (14%) to purified Cuc m 3. This discrepancy was attributed to a potential monovalency and/or low IgE-binding affinity. IgE-binding to Cuc m 3 reflected 40 and 70% of the IgE reactivity to extract from pulp and juice, respectively. In 2014, the full cDNA sequence of a muskmelon PR-1 protein derived from the inner layer of the fruit was cloned (GenBank# ACB45874), but never included to the World Health Organization and International Union of Immunological Societies (WHO/IUIS) Allergen Nomenclature database, and therefore in this review named as Cuc m PR-1. The cDNA sequence encodes for a 151 aa polypeptide with a theoretical pI of 9.47 and a predicted molecular mass of 16.97 kDa (20). Cuc m PR-1 expresses one potential glycosylation site, six cysteins, and an SCP-like extracellular protein domain (20). In addition, Cuc m PR-1 shows aa sequence identities with Cyn d 24 (42.6%), and other PR-1 proteins from cucumber (70%) or tomato (61%) (Table 1), which up to now not have been described as allergens, but are discussed as potential cross-reactive allergens (20, 21).

**Table 1:** AA-identities of Pru p 9, Art v 2, Cyn d 24 and Cuc m 3, including the full length sequence of a Cuc m PR-1 protein, without including the signal peptide.

	**Peach (Pru p 9) XP0071** **99020**	**Mugwort (Art v 2) CAK50834**	**Bermuda grass (Cyn d 24) AAU15051**	**Muskmelon ACB45874**	**Muskmelon (Cuc m 3) P83834**	**Cucumber Q8S3W2**	**Grape Q7XAJ6**	**Bellpepper AAK30143**	**Tomato ACB88202**	**Potato Q9SC15**	**Rape Q43392**	**Barley Q43489**	**Maize O82086**	**Japanese hop MN971582**
Peach (Pru p 9) XP007199020	**100**	40.30	41.18	42.75	43.90	39.71	46.67	42.96	42.96	43.61	46.27	41.48	42.22	36.23
Mugwort (Art v 2) CAK50834	40.30	**100**	40.44	43.07	40.00	43.94	50.38	50.38	51.15	55.04	48.85	48.09	46.56	39.71
Bermuda grass (Cyn d 24) AAU15051	41.18	40.44	**100**	42.55	31.71	44.53	47.01	45.93	47.41	44.36	44.36	48.91	45.26	45.77
Muskmelon ACB45874	42.75	43.07	42.55	**100**	80.49	70.29	68.38	62.50	61.11	59.26	59.26	59.12	60.58	49.65
Muskmelon (Cuc m 3) P83834	43.90	40.00	31.71	80.49	**100**	60.98	68.29	53.66	53.66	55.00	53.66	56.10	63.41	41.46
Cucumber Q8S3W2	39.71	43.94	44.53	70.29	60.98	**100**	62.50	62.04	63.50	63.70	62.22	62.32	61.59	43.48
Grape Q7XAJ6	46.67	50.38	47.01	68.38	68.29	62.50	**100**	71.32	69.85	70.15	62.96	63.97	61.76	47.06
Bellpepper AAK30143	42.96	50.38	45.93	62.50	53.66	62.04	71.32	**100**	96.15	69.63	60.74	67.15	61.31	42.34
Tomato ACB88202	42.96	51.15	47.41	61.11	53.66	63.50	69.85	96.15	**100**	68.89	60.74	67.15	59.85	41.61
Potato Q9SC15	43.61	55.04	44.36	59.26	55.00	63.70	70.15	69.63	68.89	**100**	63.91	62.96	60.74	45.19
Rape Q43392	46.27	48.85	44.36	59.26	53.66	62.22	62.96	60.74	60.74	63.91	**100**	60.00	60.74	40.00
Barley Q43489	41.48	48.09	48.91	59.12	56.10	62.32	63.97	67.15	67.15	62.96	60.00	**100**	74.29	49.28
Maize O82086	42.22	46.56	45.26	60.58	63.41	61.59	61.76	61.31	59.85	60.74	60.74	74.29	**100**	48.55
Japanese hop MN971582	36.23	39.71	45.77	49.65	41.46	43.48	47.06	42.34	41.61	45.19	40.00	49.28	48.55	**100**

### Cyn d 24, a PR-1 From Bermuda Grass

Pollen from the subtropical Bermuda grass (*Cynodon dactylon*) is a major source for allergic reactions with a high prevalence of IgE-sensitization in grass pollen allergic patients, ranging from 40% up to 91% in India and Australia, respectively (22). In total, seven allergens were identified (allergen.org): Cyn d 1 (beta-expansin), Cyn d 7 (polcalcin), Cyn d 12 (profilin), Cyn d 15 and Cyn d 23, Cyn d 22 (enolase), and the PR-1 protein Cyn d 24. Shen et al. showed the abundance of an IgE-reactive 21 kDa protein in Bermuda grass pollen, with a frequency of 29% of specific IgE-binding in Bermuda grass allergic patients (23). Using a proteomic approach by 2D LC-MS/MS, N-terminal sequencing, and an anti-PR-1 antibody, the 21 kDa protein (pI = 5.9) was identified as PR-1 protein (24). Four peptides from two spots revealed 39–54% aa similarity with the corresponding region of PR-1 from *Hordeum vulgare* (P35793). Glycoprotein staining indicated a glycosylation of the PR-1 protein. Finally, natural Cyn d 24 was purified (25) and the cDNA was cloned (GenBank# AAU15051.1). Cyn d 24 consists of 153 aa with an additional signal peptide of 97 aa and it displays an SCP-like extracellular protein domain. Remarkably, Cyn d 24 reveals higher aa identities to SCP proteins than to CRISPs, but low aa-identity to group allergen 5. It contains the conserved region GHYTQVVW, which is responsible for plant defense-related activity (25). Mass spectrometry data of the purified nCyn d 24 resulted in a mass of *m/z* 18.4 kDa, The pI was estimated to be 5.9 and periodic acid Schiff staining indicated that the protein is glycosylated. Con A binding of nCyn d 24 demonstrated that it likely contains carbohydrate moieties with free α-d-mannopyranoside or α-d-glucopyranoside residues (25). Enzyme-linked immune sorbent assay (ELISA) experiments showed that 65% (23/35) of Bermuda grass pollen allergic patients were sensitized to nCyn d 24. About 5.7% of the mass of Cyn d 24 consists of the oligosaccharide Man_3_GlcNAc_2_Fuc, probably a unique feature of PR-1 glycoproteins, which leads to the hypothesis, that these glycan structures could display IgE crossreactive carbohydrates (25). Moreover, Cyn d 24 and other glycoproteins from Bermuda grass pollen carry non-xylosylated glycans, mainly MMF^3^, which are also found in insect venom (26). Unfortunately, no studies about IgE crossreactivity of Cyn d 24 with other PR-1 allergens from pollen, food, or insect venom have been described until now.

### Art v 2 and Artimisia PR-1 Allergens

*Artemisia vulgaris* (27) belongs to one of the largest families of flowering plants, the *Compositae* or *Asteraceae*. It is a weed widely spread in temperate regions and subtropics of Europe, North America, and Asia (28, 29). Among these, Art v 1, Art v 2, Art v 3, and Art v 7 have been shown to be the most relevant allergens (28, 29). However, the function of PR-1 homologous Art v 2 remains unknown (14).

The apparent molecular mass of Art v 2 on gel filtration chromatography is 33 ± 3 kDa, suggesting that it is composed of two mainly identical subunits linked by disulfide bridges (30). Purified nArt v 2 gave a single band with a molecular mass of 19.2 kDa under reduced conditions and 34.2 kDa under non-reduced conditions. It has one potential N-glycosylation site and a calculated pI of 5.23. However, in contrast to Cyn d 24, modification of high-mannose oligosaccharides has been reported to have no significant effect on the binding of IgE. Nevertheless, it could be a stabilizing element of the three-dimensional conformation (31). Art v 2 has six conserved cysteine residues that should be involved in the stability of the 3D structure (27). This protein folds into a sandwich structure with four α-helices and three ß-strands arranged antiparallelly and compacted by three disulfide bridges (Figure 1B) (31, 32).

Art v 2 homologous allergen sequences in *Artemisia* spp. are highly conserved, indicating a general crossreactivity in all species of this genus (27). In this way, a sequence identity of 40% was found with the allergen Cyn d 24 from Bermuda grass pollen (Table 1). In addition, Art v 2 shares between 47 and 55% aa-identity with PR-1 proteins from tomato (51%), potato (55%), or bell pepper (50%) between others (Table 1) (30), with unknown allergenic properties till now.

Determination of Art v 2-specific IgE-values showed that 63.2% of sera from mugwort-allergic patients reacted with nArt v 2, whereas 10.5% reacted with the recombinant counterpart. The difference in reactivity between the natural and the recombinant allergen is attributed to the lack of the *N*-glycosylation reported in the nArt v 2 (30). Regarding skin testing, the prevalence of nArt v 2 sensitization was 57.9% at 100 μg/ml and 36.8% at 50 μg/ml, whereas no false positive was detected among the control patients. A positive skin prick test using rArt v 2 was determined only in 21% of the allergic patients (33).

Artemisia pollens have been recognized as a major cause for seasonal allergic respiratory disease worldwide, especially asthma (28, 34). It is relevant along with the Asia–Europe silk-road and in northwestern European countries. Besides, Egger et al. have described the possible role of Art v 2-like protein on pollen food syndromes associated with mugwort pollinosis (32).

However, the information on other PR-1 related proteins in this plant family is limited since studies have been carried out mainly with *Artemisia vulgaris*. As an example, *Artemisia annua* (Art an 2) has been recognized as an important source of allergen, and together with *Artemisia argyi* (Art ar 2) and *Artemisia sieversiana* (Art si 2), they are recommended for immunotherapy in Chinese allergic patients (29).

### Pru p 9, a PR-1 From Peach

Peach tree (*Prunus persica*) allergens are present in fruit, pollen, leaves, and branches, and can induce systemic, respiratory, gastrointestinal, and cutaneous symptoms. Although classified as entomophilous, like other pollens, recent evidence indicates that in areas with peach tree orchards, pollen grains can be transported by the wind for short distances, facilitated by the presence of pollutants or the disruption of pollen in smaller submicronic particles (35, 36). In addition, in these areas, there is a high prevalence of sensitization and allergy to peach tree pollen (37).

Six peach allergens that are mostly involved in food allergy have been identified and characterized: Pru p 1 (PR-10 protein) (38), Pru p 2 (Thaumatin-like), Pru p 3 (ns-LTP) (39), Pru p 4 (profilin) (40), Pru p 5 (Hev b 5-like) (41), and Pru p 7 (gibberellin-regulated protein) (42). On the other hand, the only aeroallergen described in peach tree pollen is Pru p 9 (GenBank# XP_007199020.1), a PR-1 protein (43).

In a study analyzing 685 children and adolescents between 3 and 19 years and 310 adults from the population, directly or indirectly exposed to peach cultivars, the sensitization to peach tree pollen was estimated to be 20%, being the third most prevalent pollen after olive tree (33%) and grass pollen (26%) (43, 44). According to symptoms, conjunctivitis appeared in 47% of the cases, rhinitis in 42%, and asthma in 14% (43).

Pru p 9 is a 138 aa polypeptide with a theoretical pI of 5.11 and a molecular mass of 18 kDa. This allergen is responsible for the induction of respiratory and ocular symptoms in allergic subjects, independently whether they are directly or indirectly exposed. Reported in another study by Blanca et al., from 62 cases sensitized to peach tree pollen, 41% were positive in SPT to Pru p 9 and presented Pru p 9-specific IgE-antibodies. Besides, the nasal provocation tests showed a decrease in the nasal cavity volume greater than 20% with the new aeroallergen described (42), associated with rhinorrhoea, sneezing, nasal itching, eye redness, and tearing. In addition, Victorio-Puche et al. (45) described the implication of Pru p 9 in the induction of occupational allergic respiratory symptoms, with rhinitis and asthma in 43% of workers in the orchard after handling peach trees during the flowering period. Unfortunately, no studies about IgE crossreactivity of Pru p 9 with other PR-1 allergens from pollen or food (including peach fruit) have been described till now.

### Vesp ma 5

Stings of hymenoptera are one of the most frequent triggers for severe IgE-mediated anaphylaxis in adults. Noteworthy, PR-1 proteins share similar features with antigen 5 (Ag5) allergens from the venom of hymenoptera, such as the CAP domain forming a potential CAP cavity for ligand binding. Ag5 showed only moderate aa-ids with Pru p 9: 18% with horsefly Ag5 (Tab y 5), 24% with Savannah tsetse fly Ag5 (Glo m 5), and up to 35% with Vesp ma 5. The Ag5 protein family includes several allergens from hornets, wasps, horseflies, and fire ants: The 25 kDa wasp *Vespa magnifica* Vesp ma 5 (46) shares 27–87% aa-id with Tab y 5 (*Tabanus yao*) (47), Vesp m 5 (*Vespula maculifrons*) (48), Ves v 5 (*Vespula vulgaris*) (49), Dol m 5 (*Dolichovespula maculata*) (50), and Vesp c 5 (*Vespa crabro*) (51), respectively. To date, 26 *Vespoidea* Ag5 proteins are listed in the official allergen nomenclature database of the WHO/IUIS Allergen Nomenclature database (52). According to the phylogenetic distance between these species, the Ag5 allergens exhibit a varying degree of sequence identity and, therefore, most likely of crossreactivity between them.

Although Ag5 represents one of the most abundant and important major venom allergens in almost all allergy-relevant *Vespoidea* species (51, 53–56), their function within the venoms remains widely unclear. In blood-feeding ticks, flies, and mosquitoes, Ag5 proteins are salivary proteins that are thought to function either in suppression of the host immune system or in preventing platelet aggregation (57). The presence of Ag5 allergens (as well as hyaluronidases), which exhibit crossreactivity with their homologs of wasp venom, in the saliva of horseflies and mosquitoes (47), may explain the postulated “wasp–mosquito–horsefly-syndrome,” in which wasp venom-allergic patients also experience systemic reactions after bites of mosquitoes or horseflies (58, 59). Due to their outstanding role as major allergens, Ag5 proteins build a key element for the diagnosis of *Vespoidea* venom allergy (60).

### Other Likely Allergenic PR-1 Proteins

The IgE-sensitizing capacity has been shown for additional PR-1 proteins. However, these proteins have not yet been fully characterized and are not included in the IUIS allergen database.

Feverfew *(Parthenium hysterophorus)*, an invasive weed from the *Asteraceae* family, has been reported as a respiratory allergen. Recently, a pollen-derived PR-1 protein of 20 kDa, which displays IgE-reactivity with sera from feverfew sensitized patients' from India, has been identified by LC-MS/MS and 2D-LC-MS/MS (61). However, the molecular characterization of Par h PR-1 is lacking.

Moreover, screening a cDNA expression library revealed that a PR-1 protein from Japanese hop (*Humulus japonicus*), an important cause of weed pollinosis in East Asia, is recognized by IgE from 3.4% (1/29) of Japanese hop allergic patients with a positive case history and IgE-sensitization to Japanese hop (62). Hum j PR-1 encodes for a 171 aa peptide (apparent MW of 20 kDa) and shares 40–46% of aa-sequence identity with Art v 2, Cuc m 3, and Cyn d 24 (Table 1). The authors attributed the low IgE-binding frequency of the His-tagged protein with a potential engagement of glycan moieties in IgE-epitopes of the natural counterpart and/or an incorrect protein folding affecting conformational epitopes.

Finally, a 20 kDa IgE-reactive protein derived from tomato pollen has been described to play the role as an occupational allergen for a greenhouse worker (63). Initial experiments showed partial IgE-crossreactivity with purified Pru p 9 (Pedraza et al. unpublished data).

## Conclusions

Pathogenesis-related proteins are characterized by certain common biochemical features, such as low molecular weight, stability at low pH, and resistance to proteases. This protein family is widely distributed in mono- and dicotyledonous species (64). Members of the SCP superfamily have been proposed to act in the defense against pathogens as Ca^++^-chelating serine proteases (3). The Ca^++^-chelating function would fit with various signaling processes that members of this family, such as the CRISPs, are involved in, supported by sequence and structural evidence of a conserved pocket containing two histidine and glutamate. In plants, PR-1 proteins are among the most abundantly produced proteins during defense responses and have been reported to constitute 2% of the total leaf protein in pathogen-infected tobacco (65). PR-1 proteins are typically 14–17 kDa polypeptides of approximately 135 aa that form disulfide bonds and a compact 3D structure, conferring the protein a high stability (14). In recent years, a variety of PR-1 proteins and their homologs causing allergenicity have been characterized. The size, stability, and resistance to proteases along with hydrolytic and membrane permeabilizing ability make these proteins excellent candidates to elicit allergenic responses (1, 2, 15, 66). However, very little is known about their allergenic properties (4).

The PR-1 proteins from plants and other organisms share structural similarities that may trigger crossreactivity reactions (67). Evident aa-identities within the PR-1 proteins likely could lead to IgE-crossreactivity and even to the presence of further allergenic PR-1 proteins, in e.g., tomato, potato, rape, or maize. Moreover, particularly interesting in the area of allergy is their relationship (approximately 30% of sequence identity) with Ag5 allergens (14), but no studies have been performed yet concerning this.

The effect of posttranslational modification of PR-1 proteins by carbohydrate determinants needs to be further explored. Pru p 9 contains two potential glycosylation sites at aa_24_ and aa_52_, whereas Art v 2 (aa_91_), Cuc m PR-1 (aa_33_), and Cyn d 24 (aa_25_) have only one potential glycosylation site, as predicted using the NetNGlyc – 1.0 server (68). Interestingly the N-glycosylation sites of Pru p 9 (aa_52_), Cyn d 24 and Cuc m PR-1 are conserved between their AA sequence and therefore, could be influencing or be responsible for IgE crossreactivity reactions through crossreactive carbohydrate determinants (25). Besides Cyn d 24 other glycoproteins from Bermuda grass pollen carry non-xylosylated glycans, mainly MMF which are also found in insect venom (26). Similarly, the predicted legume PR-1 protein showed better binding affinity to carbohydrates (glycans) than proteins/lipids, indicating that glycans might act as recognition clues or trigger immune responses in the plant (4).

The clinical relevance of the PR-1 family is partially known. Only few allergens from this widely distributed protein family have been deeply studied, such as Cyn d 24 with 63% of recognition by patients allergic to Bermuda grass pollen and Art v 2 with 63.2% of positive response by patients sensitized to mugwort pollen, or Cuc m 3, a PR-1 food allergen identified in melon allergic patients. In a more complex way, the Ag5 group, encompassing 26 PR-1 allergens, is implicated in hymenoptera allergy, which triggers life-threatening reactions. According to WHO/IUIS Allergen Nomenclature database criteria, all of them could be considered as relevant allergens in their corresponding sources. An important feature of PR-1 proteins is their role as occupational allergens. Of particular interest is the PR-1 protein described in peach tree pollen, Pru p 9, with 43% of IgE binding in patients allergic to this source. In this way, Pru p 9 has been identified as a relevant allergen with critical implications for people working with peach tree cultivars, developing respiratory symptoms (45).

Nevertheless, one of the major difficulties encountered by researchers in studying allergens belonging to the PR-1 protein family lies in the difficulty for identifying cases sensitized, and also for obtaining pollen grains for molecular analysis of their allergenic profile. Probably, this is one reason for the low number of PR-1 allergens identified and registered in databases. However, it is very likely that PR-1 proteins from other sources display allergenic properties. Exploring new PR-1 proteins implicated in allergenicity, and a complete understanding of their structures and IgE-binding epitopes are necessary for improving the diagnoses and treatment in these subjects, reaching precision medicine in allergy.

## Author Contributions

LM-P, AW, and SS conceived and supervised the review topics. LM-P, AW, SS, MB, MS, and NB-L wrote the first draft. All authors contributed to the article and approved the submitted version.

## Conflict of Interest

The authors declare that the research was conducted in the absence of any commercial or financial relationships that could be construed as a potential conflict of interest.

## Publisher's Note

All claims expressed in this article are solely those of the authors and do not necessarily represent those of their affiliated organizations, or those of the publisher, the editors and the reviewers. Any product that may be evaluated in this article, or claim that may be made by its manufacturer, is not guaranteed or endorsed by the publisher.

## Abbreviations

PR: pathogenesis-related
CRISP: cysteine-rich secretory proteins
Ag5: antigen 5
SCP: Sperm Coating Protein
TAPS: Tpx-1/Ag5/PR-1/Sc7
CAP: Cysteine-rich secretory proteins
Antigen 5: PR-1
Aa: amino acid
DBPCFC: Double-blind placebo-controlled food challenge.
